# Neuroimaging advances regarding subjective cognitive decline in preclinical Alzheimer’s disease

**DOI:** 10.1186/s13024-020-00395-3

**Published:** 2020-09-22

**Authors:** Xiaoqi Wang, Weijie Huang, Li Su, Yue Xing, Frank Jessen, Yu Sun, Ni Shu, Ying Han

**Affiliations:** 1 Department of Neurology, Xuanwu Hospital of Capital Medical University, Beijing, 100053 China; 2 Center of Alzheimer’s Disease, Beijing Institute for Brain Disorders, Beijing, China; 3 State Key Laboratory of Cognitive Neuroscience and Learning & IDG/McGovern Institute for Brain Research, Beijing Normal University, Beijing, 100875 China; 4 Center for Collaboration and Innovation in Brain and Learning Sciences, Beijing Normal University, Beijing, China; 5 Beijing Key Laboratory of Brain Imaging and Connectomics, Beijing Normal University, Beijing, China; 6 Department of Psychiatry, University of Cambridge, Cambridge, UK; 7 Sino-Britain Centre for Cognition and Ageing Research, Southwest University, Chongqing, China; 8 Radiological Sciences, Division of Clinical Neuroscience, University of Nottingham, Nottingham, UK; 9 Department of Psychiatry and Psychotherapy, Medical Faculty, University of Cologne, 50937 Cologne, Germany; 10 German Center for Neurodegenerative Diseases (DZNE), Bonn, Germany; 11 Excellence Cluster on Cellular Stress Responses in Aging-Associated Diseases (CECAD), University of Cologne, Cologne, Germany; 12 National Clinical Research Center for Geriatric Disorders, Beijing, China

**Keywords:** subjective cognitive decline, Alzheimer’s disease, neuroimaging, multimodal MRI, PET

## Abstract

Subjective cognitive decline (SCD) is regarded as the first clinical manifestation in the Alzheimer’s disease (AD) continuum. Investigating populations with SCD is important for understanding the early pathological mechanisms of AD and identifying SCD-related biomarkers, which are critical for the early detection of AD. With the advent of advanced neuroimaging techniques, such as positron emission tomography (PET) and magnetic resonance imaging (MRI), accumulating evidence has revealed structural and functional brain alterations related to the symptoms of SCD. In this review, we summarize the main imaging features and key findings regarding SCD related to AD, from local and regional data to connectivity-based imaging measures, with the aim of delineating a multimodal imaging signature of SCD due to AD. Additionally, the interaction of SCD with other risk factors for dementia due to AD, such as age and the *Apolipoprotein E* (*ApoE*) ɛ4 status, has also been described. Finally, the possible explanations for the inconsistent and heterogeneous neuroimaging findings observed in individuals with SCD are discussed, along with future directions. Overall, the literature reveals a preferential vulnerability of AD signature regions in SCD in the context of AD, supporting the notion that individuals with SCD share a similar pattern of brain alterations with patients with mild cognitive impairment (MCI) and dementia due to AD. We conclude that these neuroimaging techniques, particularly multimodal neuroimaging techniques, have great potential for identifying the underlying pathological alterations associated with SCD. More longitudinal studies with larger sample sizes combined with more advanced imaging modeling approaches such as artificial intelligence are still warranted to establish their clinical utility.

## Highlights

- The main imaging features and neuroimaging advances in individuals with SCD related to AD are summarized.
- The symptoms of SCD are associated with specific and distinctive underlying pathological events.
- A preferential vulnerability of AD-signature regions in individuals with SCD are described.
- The risk factors for dementia due to AD may interact with SCD and aggregate brain alterations.

## Background

Alzheimer’s disease (AD) is one of the most common causes of dementia, with a dramatically increasing incidence in recent years that is expected to reach 115 million in 2050 [World Alzheimer Report 2018, www.alz.co.uk]. Currently available treatments are moderately beneficial at best for the symptomatic stages of AD [1]. Patients with preclinical AD are defined as cognitively unimpaired individuals with abnormal AD biomarkers [2]. At this very early stage, individuals still have sufficiently intact cognitive function that can be harnessed and directed toward either compensation or restitution of function [3]. More importantly, evolving biomarker studies enable to identify individuals with preclinical AD, which provides the best opportunity for therapeutic success and prevents cognitive decline before the onset of clinical symptoms.

Subjective cognitive decline (SCD), the first clinical manifestation in the AD continuum, is self-experienced decline in cognitive function without evidence of objective cognitive impairment [3–5]. SCD is postulated to manifest at a relatively late phase of preclinical AD and is associated with increased risks of AD biomarker abnormalities and future cognitive decline and dementia [2, 4, 6], making it a high-risk condition for the development of dementia due to AD. However, knowledge about the relationship between SCD and AD neuroimaging biomarkers is still controversial. Thus, investigating populations with SCD is important for understanding the early pathological mechanisms of preclinical AD and identifying SCD-related biomarkers, which are crucial for the early detection of AD with relatively inexpensive and easy measures.

Although SCD is a heterogeneous concept that can be induced by many conditions other than AD [4], including normal aging, psychiatric conditions (e.g., depression), other neurological and medical disorders (e.g., frontal temporal dementia and dementia with Lewy bodies), substance abuse and certain medications, we specifically focus on SCD due to AD, and these other conditions have been excluded from all studies included in this review. With the advent of neuroimaging techniques, such as positron emission tomography (PET) and magnetic resonance imaging (MRI) [7], structural and functional brain alterations have been detected *in vivo* during the asymptomatic stage of AD [8, 9]. An increasing number of neuroimaging studies have indicated that the symptoms of SCD are associated with specific and distinctive underlying pathological events, such as the abnormal deposition of ß-amyloid and tau proteins, gray matter atrophy, disruptions in the white matter (WM) and deficits in brain function [3, 10, 11]. The utility of neuroimaging techniques makes it possible to understand the neuropathological mechanisms underlying SCD related to AD and to provide potential pathological and imaging biomarkers for the early detection and even prediction of AD.

The purpose of this review is to provide a state-of-the-art and comprehensive summary of the literature regarding advances in neuroimaging findings in individuals with SCD within the context of AD by including studies using different imaging modalities. The limitations of current studies and future directions are also discussed.

## Methods

**Figure 1:**
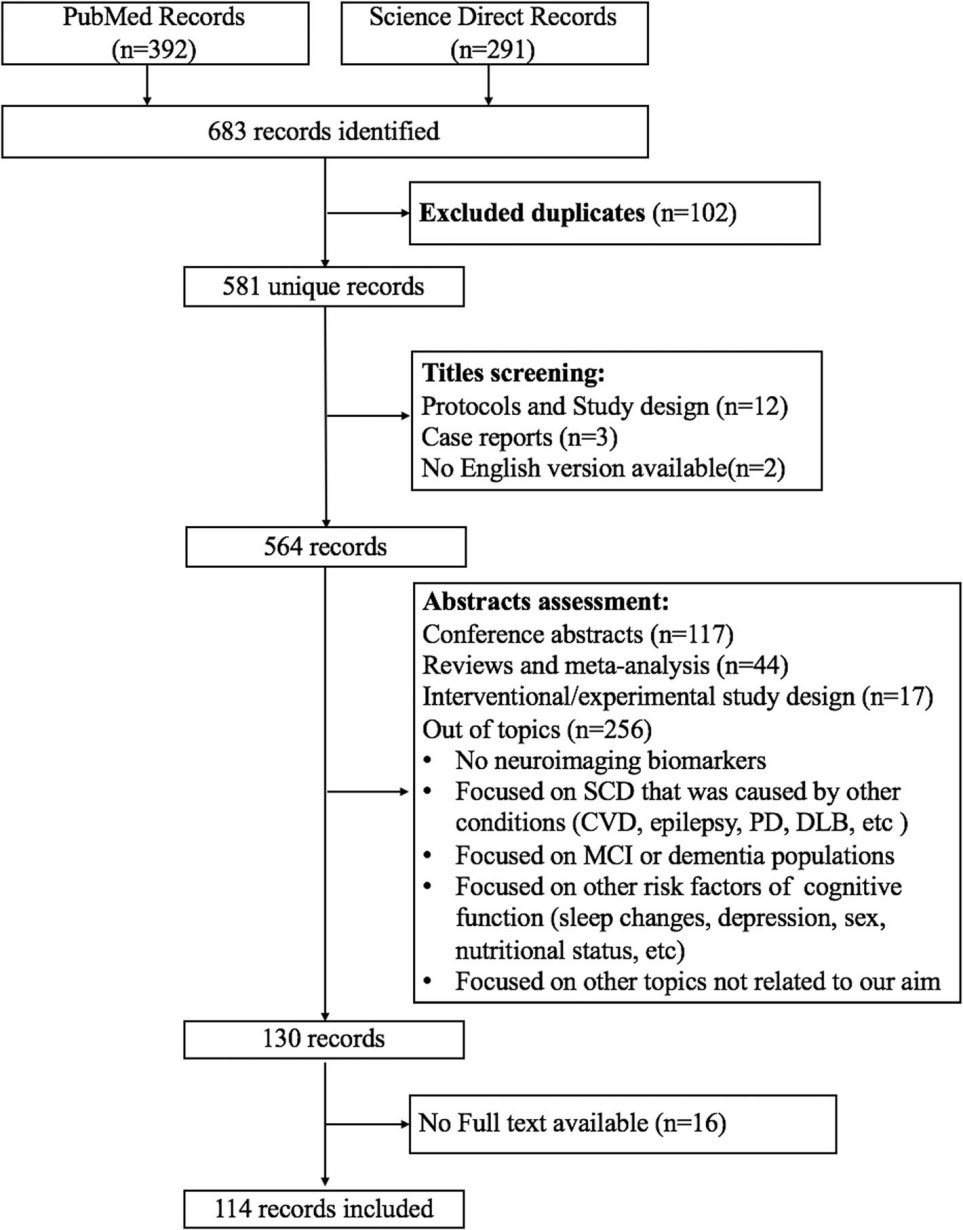
Flowchart of the literature screening process and results. Abbreviations: SCD=subjective cognitive decline; CVD=cerebrovascular disease; PD=Parkinson’s disease; DLB=dementia with Lewy body; MCI=mild cognitive impairment; AD=Alzheimer’s disease

We searched the PubMed and Science Direct databases for articles describing the neuroimaging changes in individuals with SCD related to AD published from January 1994 to September 2019. The search terms used were “((subjective cognitive decline[Title/Abstract]) OR (subjective memory decline[Title/Abstract]) OR (subjective cognitive complaint[Title/Abstract]) OR (subjective cognitive complaints[Title/Abstract]) OR (subjective memory impairment[Title/Abstract]) OR (subjective memory impairments[Title/Abstract]) OR (subjective memory complaint[Title/Abstract]) OR (subjective memory complaints[Title/Abstract]) OR (cognitive complaints[Title/Abstract]) OR (subjective cognitive impairment[Title/Abstract]) OR (subjective cognitive impairments[Title/Abstract])) AND (Alzheimer’s disease) AND (neuroimaging OR PET OR MRI OR EEG OR MEG OR NIRs OR ASL OR DKI OR DSI OR DTI OR fMRI OR sMRI OR QSM)”. The studies were included based on the following inclusion criteria: (1) studies that described neuroimaging changes in individuals with SCD related to AD, (2) participants with SCD exhibited normal performance on standard neuropsychological tests and were free of other medical or psychiatric causes, and (3) original research published in English with the full-text available, regardless of the research settings. The following types of studies were excluded: (1) case reports, conference abstracts, reviews and study design or protocols, (2) studies with an interventional/experimental study design, and (3) studies not related to our topics: i.e., studies that did not use neuroimaging markers and studies focused on SCD that was caused by other conditions (e.g., cerebrovascular disease, epilepsy, Parkinson’s disease, dementia with Lewy bodies, etc.), studies focused on MCI or dementia populations, studies focused on other risk factors for impaired cognitive function (e.g., sleep changes, depression, nutritional status, etc.) and studies focused on other topics not related to our aim. After detailed evaluations, 114 studies were included and reviewed. A detailed description of the article selection process is presented in the flowchart (Fig. 1). We discussed and summarized the neuroimaging changes related to SCD in the background of AD in detail according to the different neuroimaging modalities, including neuroimaging performed at the molecular, structural, and functional levels.

## Standard terminology and diagnostic criteria

The concept of SCD was first introduced in the early 1980s by Reisberg and colleagues to define an early stage of AD and was initially assessed using the Global Deterioration Scale [12]. In recent years, SCD has received various labels, including subjective cognitive complaints, subjective memory complaints, subjective cognitive impairment, subjective memory impairment, subjective memory decline and others [4]. Although the impairment has attracted increasing attention since 2005, SCD research has been hampered by the lack of a common nomenclature, and uniform criteria for defining SCD are not available. Some studies identified SCD with a single question such as “Do you feel you can remember things as well as you used to?”, while other groups assessed SCD with structured questionnaires such as the Mood and Feelings Questionnaire (MFQ) and/or the self-evaluated Everyday Cognition Scale (E-Cog). In 2014, a consensus terminology and a conceptual framework for research on SCD related to AD was proposed by the Subjective Cognitive Decline Initiative (SCD-I) [4]. This framework unified the multiple descriptors into a single term, SCD, and presented a set of features of SCD due to AD, named “SCD-plus”, to facilitate the comparison of study findings, data pooling, meta-analyses, and collaborative multicenter research. These features include onset within 5 years, age at onset ≥ 60 years, concerns regarding feelings of worse performance than other individuals of the same age, confirmation of cognitive decline by an informant, possession of the *Apolipoprotein E* (*ApoE*) ɛ4 genotype and the presence of biomarker evidence for AD. Recently, two additional SCD plus features were proposed, including consistent SCD over time and seeking medical help because of SCD [5]. The framework also supports some flexibility in the classification of SCD, but researchers are responsible for explicitly clarifying how they operationalize SCD and why they chose a particular approach. All these efforts help further advance the investigation of SCD related to AD.

Only a few longitudinal studies of people with carefully phenotyped SCD have investigated the rate of progression of SCD to dementia due to AD [13, 14]. With clinical advances, the increasing number of memory clinics will facilitate the enrollment of subjects with SCD and increase awareness about SCD and the concept of cognitive protection.

## Positron emission tomography

### β-amyloid deposition

The advent of PET amyloid tracers allows the quantification of amyloid deposition *in vivo* [15–17]. ^11^C-Pittsburgh Compound B (PiB) was the first amyloid PET ligand with high affinity for amyloid [18]. Additionally, the development of ^18^F-labeled ligands (^18^F-florbetapir, ^18^F-florbetaben and ^18^F-flutemetamol) has facilitated their widespread use due to longer half-lives (~110 min) [19–21]. A meta-analysis reported a prevalence of amyloid pathology among individuals with SCD of 12%-43% [22]. According to Perrotin et al. and Snitz et al., subjects with SCD showed a significant increase in amyloid positivity compared with healthy controls (HCs) [23, 24]. Furthermore, several studies using the amyloid load as a continuous variable verified the relationship between the amyloid burden and the severity of SCD [25–29]. However, some other studies did not report this relationship [30–33]. Therefore, the correlations between the existence and severity of SCD with amyloid pathology remain inconsistent, potentially due to the different research settings and methods for the operationalization of SCD used among studies.

In clinical patients with dementia due to AD, amyloid deposition exhibits a diffuse pattern that initiates from the prefrontal and posterior parietal regions with the highest vulnerability [34, 35]. Interestingly, a similar pattern of the spatial distribution of amyloid deposition was also observed in individuals with SCD, which was mainly distributed in the temporal [24], medial prefrontal, anterior and posterior cingulate cortices and the precuneus [25]. Therefore, consistent with the “amyloid cascade” hypothesis [36], the increase in amyloid levels in AD-specific regions might be associated with SCD, potentially more than 10 years prior to the ensuing cognitive impairment.

Furthermore, several studies have focused on the clinical features of SCD, such as specific involved cognitive domains, related concerns, and the confirmation of informants, and explored their relationships with the amyloid burden to increase the predictive power of SCD to the underlying amyloid pathology. For example, Amariglio et al. (2012) revealed a significant correlation between scores of the memory and executive subscales of SCD with the amyloid burden [29]. As shown in another study, self-reported confusion, but not the total SCD score, predicted global and regional amyloid deposition in SCD [32]. Moreover, SCD-related worry was also found to be associated with the cortical amyloid load [33]. Therefore, the additional features of SCD may enhance its association with AD pathology.

Recently, several studies have consistently suggested that higher amyloid aggregation at baseline indicated a faster rate of decline in various cognition domains [37, 38] and a more rapid increase in SCD severity over time [39] in individuals with SCD. In addition, subjects in the amyloid-positive group with a greater number of subjective memory complaints displayed a greater rate of progression to mild cognitive decline (MCI) [40] and dementia due to AD [41]. These investigations provided evidence that SCD with a high amyloid load may indicate a faster longitudinal cognitive decline and worse clinical progression.

Notably, amyloid deposition in subjects with SCD is also affected by other risk factors for dementia due to AD, such as the *ApoE* ɛ4 genotype and age. Individuals with SCD may present higher allelic frequencies of *ApoE* ɛ4, and cerebral amyloid levels might be partially predicted by the *ApoE* ɛ4 level [42]. In another study of individuals with SCD from the Alzheimer’s Disease Neuroimaging Initiative (ADNI) database, *ApoE* ɛ4 carriers with SCD showed higher levels of amyloid accumulation than noncarriers. However, no hypometabolism or atrophy was detected [43]. Aging is also associated with increased cerebral amyloid deposition in cognitively normal elderly people. A meta-analysis [22] reported an association between the occurrence of cerebral amyloid pathology in cognitively normal individuals and age, increasing from 10% to 44% in individuals between 50 and 90 years of age. Zwan et al. (2016) assessed multiple risk factors for high amyloid levels, including SCD, the *ApoE* genotype, age, and episodic memory [44], to further elucidate the relationship. However, the effects of the interactions among SCD, the *ApoE* genotype, age and other potential contributing factors on preclinical AD are complicated and are not completely understood to date. Therefore, additional investigations are needed to clarify the relationship among different risk factors for dementia due to AD.

### Tau burden

Pathological tau deposition represents another key biomarker of AD [45, 46]. The development of selective tau tracers, such as the most extensively used tracer ^18^F-Flortaucipir [47], allows researchers to investigate regional distribution of tau pathology in the living human brain [46, 48, 49]. In the preclinical stage, the relationship between the symptoms of SCD and tau burden has been reported by Swinford and colleagues [50]. They scanned SCD subjects using ^18^F-flortaucipir PET and determined that the self-reported memory concern was associated with frontal tau levels, whereas the informant-reported concern was related to parietal tau levels. However, until now, the tau PET studies in individuals with SCD are currently limited. More evidence is needed to further elucidate the association between tau and amyloid pathology and their contribution to SCD and clinical progression.

### Cerebral glucose metabolism

^18^F-Fluorodeoxyglucose (FDG)-PET has been used to quantify abnormal cerebral glucose metabolism [40, 51, 52]. Hypometabolism in individuals with MCI and dementia due to AD have been consistently confirmed, primarily in the posterior parietal and temporal regions [53, 54]. The hypometabolic profile in AD-related regions has been characterized in subjects with SCD and involves the parietotemporal cortex, precuneus, precentral and parahippocampal gyri [55–57]. However, Scheef et al. (2012) observed increased metabolism in the right medial temporal lobe in the SCD group compared with HCs, which may suggest compensatory neuronal activity. Additionally, metabolic deficits associated with SCD have been observed in high-risk genetic carriers of *ApoE* ɛ4 [55].

Moreover, some studies have explored the relationship between regional glucose metabolism and cognitive performance in subjects with SCD. For example, the metabolism in both the left hippocampus and the right amygdala was positively correlated with verbal logical memory immediate recall [58]. FDG-PET not only enables the prediction of cognitive function but also may serve as a prognostic marker for tracking longitudinal cognitive changes. Scheef and colleagues identified an association between hypometabolism in the right precuneus at baseline and the degree of longitudinal memory decline [59]. Based on these findings, hypometabolism may be a potential functional biomarker for the early detection of AD (Table 1).

**Table 1:** Summary of PET studies

Authors	Definition of SCD	Modality	Design	Sample (mean age ± SD)	Main findings
Amariglio et al. (2012) [29]	E-Cog; MFQ; Composite of 7 questions	PiB-PET	Cross-sectional	SCC: n=131(73.5±6.0) amyloid-: n=97(72.7±5.9) amyloid+: n=34(75.5±6.9)	SCC score relate to cortical PiB binding
Amariglio et al. (2018) [39]	Composite of 7 questions	PiB-PET	Cross-sectional	All: n=279(73.4±6.1) amyloid-: n=209(72.9±6.0) amyloid+: n=70(70.0±5.7)	Amyloid positivity individuals have pronounced progression of SCD
Buckley et al. (2016) [41]	MAC-Q scale	PiB-PET ^18^F-florbetapir PET ^18^F-flutematamol-PET MRI	Cross-sectional	NC: n=288 amyloid-: n=230(69±5.9) amyloid+: n=58(72±7.2)	High SMD related to greater rates of clinical progression, greater depressive symptom and smaller left hippocampal volume
Cacciamani et al. (2017) [31]	Composite of 15 questions IQCD ASC AD-NOS	^18^F-florbetapir PET MRI FDG-PET	Cross-sectional	High awareness: n=86(76.08±0.36) Low awareness: n=19(76.11±0.82)	No relationship between SCD score and neuroimaging markers; higher amyloid burden and lower cortical metabolism in “high awareness” group
Chen et al. (2019) [27]	Metamemory in Adulthood questionnaire	^18^F-florbetapir PET MRI	Cross-sectional	Total: n=85(66.97±15.11) Negative: n=53(61.25±14.86) Positive: n=32(76.46±9.96)	Poor memory performance mediates the relationship between amyloid and SCD
Hollands et al. (2015) [37]	MAC-Q Composite of 16 questions	PiB-PET	Cross-sectional	Low Aß: n=224(68.37±5.88) High Aß: n=65(73.46±7.33)	High Aß group show moderate decline in learning and working memory over 18 months.
McCluskey.et al. (2018) [32]	MAC-Q 1 binary question	^18^F-florbetaben PET	Cross-sectional	All: n=112(69.2, 2.5)	Self-reported confusion predicted higher global amyloid burden and regional amyloid in the prefrontal, posterior cingulate, precuneus and the lateral temporal.
Moreno–Grau et al. (2018) [42]	Cognitive complaints	^18^F-florbetaben PET ^18^F-florbetapir PET	Cross-sectional	ADNI_NC: n=182(73.4±6.3) ADNI_SMC: n=103(72.2±5.6) ADNI_EMCI: n=303(71.3±7.4) ADNI_LMCI: n=157(72.2±7.5) ADNI_AD: n=144(74.4±8.1) FACEHBI_SCD: n=200(65.8±7.1) ADNI_NC: n=182(73.4±6.3)	Higher *ApoE* ɛ4 carrier in SCD and *ApoE* ɛ4 dosage explained 9% and 11% cerebral amyloid variation.
Perrotin et al. (2017) [23]	Composite of 26 questions	^18^F-florbetapir PET MRI	Cross-sectional	Controls: n=35(65.6±8.6) SCDcommunity: n=35(70.8±7.5) SCDclinic: n=28(67.6±7.7)	Both groups with high self-reported difficulties has higher amyloid deposition
Perrotin et al. (2012) [25]	2 questions	PiB-PET MRI	Cross-sectional	High PiB uptake: n=11(75.73±6.05) Low PiB uptake: n=28(71.89±5.45)	Correlation between memory self-reports and regional PiB uptake in right medial prefrontal, anterior cingulate, right precuneus and posterior cingulate.
Risacher et al. (2015) [43]	CCI E-Cog	^18^F-florbetapir PET FDG-PET MRI	Cross-sectional	NC *ApoE* ɛ4-: n=132(73.7±6.1) NC *ApoE* ɛ4+: n=53(71.8±6.4) SMC *ApoE* ɛ4-: n=71(72.5±5.7) SMC *ApoE* ɛ4+: n=33(70.3±5.2) EMCI *ApoE* ɛ4-: n=174(71.6±7.3) EMCI *ApoE* ɛ4+: n=131(70.0±7.5)	SMC *ApoE* ɛ4+ show greater amyloid deposition than SMC *ApoE* ɛ4-
Rodda et al. (2010) [30]	Memory complain	PiB-PET	Cross-sectional	No presented	No difference in amyloid load between SCI and controls
Rowe et al. (2010) [28]	1 binary question	PiB-PET MRI		HC: n=177(71.6±7.4) MCI: n=57(75.5±7.5) AD: n=53(72.6±8.9) HC nMC: n=81(72.0±7.5) HC SMC: n=96(71.2±7.4)	SMC related to elevated PiB in *ApoE* ɛ4 carriers
Snitz et al. (2015) [24, 26]	MFQ CFQ SCCS	PiB-PET	Cross-sectional	SCD: n=14(68.1±4.0) NC: n=84(73.6±5.8)	57% of SCD and 31% of NC were PiB-positive. SCD had higher PiB retention in frontal cortex, lateral temporal cortex, and parietal cortex.
Snitz et al. (2015) [24, 26]	MFQ CFQ SCCS	PiB-PET	Cross-sectional	Total: n=92(81.2±8.4)	MFQ score relate to global PiB retention
Timmers et al. (2019) [38]	Memory clinic consultation Intact cognition	^18^F-florbetapir PET	Cross-sectional	Total: n=107(64±8)	Higher ^18^F-florbetapir BP_ND_ relates to steeper rate of decline on memory, attention/executive and language
Verfaillie et al. (2019) [33]	CCI SCF Composite of 4 questions	^18^F-florbetapir PET	Cross-sectional	Total: n=106(63.83±7.65)	Higher cortical amyloid deposition relates to SCD-related worries and higher memory deficit awareness but not to SCD questionnaires
Zwan et al. (2016) [44]	MAC-Q IQCODE-S	PiB-PET ^18^F-flutematamol PET	Cross-sectional	Low amyloid burden: n=229(71.9±6.5) High amyloid burden: n=78(75.0±7.2)	SMC with younger age and *ApoE* ɛ4 carriers had higher amyloid burden.
Swinford et al. (2018) [50]	E-Cog	^18^F-flortaucipir PET	Cross-sectional	CN: n=40(76.48±7.211) SMC: n=11(71.55±5.11) EMCI: n=31(75.32±7.29)	Memory concern and the self-perception relate to tau aggregation.
Cavedo et al. (2018) [60]	Memory complaints	^18^F-flortaucipir -PET FDG-PET MRI	Cross-sectional	Women: n=201(76.02±3.24) Men: n=117(76.05±3.85)	Men had higher amyloid load glucose hypometabolism and lower RSFC.
Gardener et al. (2016) [58]	1 binary question	FDG-PET	Cross-sectional	All: n=43(66±10.1) Non-SMC: n=23(66±8.9) SMC: n=20 (68±11.4)	Positive association between memory immediate recall and FDG-PET SUVR in the right amygdala in SMC individuals.
Matias-Guiu et al. (2017) [61]	Memory complaint	FDG-PET	Cross-sectional	HC: n=20(65.0±10.6) SMC: n=9(72.4±10.6)	FCSRT positively correlate with metabolism in the medial and anterior temporal region bilaterally, the left precuneus, and posterior cingulate; BNT results correlate with metabolism in the middle temporal, superior, fusiform, and frontal medial gyri bilaterally; VOSP results relate to the occipital and parietotemporal regions bilaterally; ToL scores correlate to metabolism in the right temporoparietal and frontal regions
Mosconi et al. (2008) [55]	Structured interview	FDG-PET	Cross-sectional	SMC- *ApoE* ɛ4-: n=7(63±5) SMC+ *ApoE* ɛ4-: n=8(60±9) SMC- *ApoE* ɛ4+: n=7(54±9) SMC+ *ApoE* ɛ4+: n=6(59±7)	*ApoE* ɛ4+ carriers had decreased CMRglc and higher CSF IP, P-Tau, T-Tau, and P-Tau/Amyloid42 levels. SMC had reduced CMRglc. *ApoE* genotype and SMC interacted on lowest PHG CMRglc and the highest CSF IP, P-Tau, and P-Tau/Amyloid42 levels.
Scheef et al. (2012) [59]	Memory clinic consultation 1 binary question	FDG-PET MRI	Cross-sectional	NC: n=56(66.4±7.2) SMI: n=31(67.6±6.2)	SMI had hypometabolism in the right precuneus and hypermetabolism in the right medial temporal lobe and gray matter atrophy in the right hippocampus. Association between longitudinal memory decline and reduced glucose metabolism in the right precuneus at baseline.
Song et al. (2016) [56]	Memory complaint	FDG-PET MRI	Cross-sectional	HC: n=42(68.02±5.44) SMI: n=31(69.94±6.44) MCI: n=47(69.55±6.65)	SMI had hypometabolism in the periventricular regions. SMI had hypometabolism in the parietal, precentral frontal, and periventricular regions.
Vannini et al. (2017) [57]	MFQ	PiB-PET FDG-PET	Cross-sectional	All: n=251(73.3±6.2) amyloid-: n=190(72.8±6.1) amyloid+: n=61(74.9±6.2)	Correlation between SMCs and FDG metabolism. SMCs interacted with amyloid burden on FDG metabolism in the bilateral medial temporal lobes.

*SCC* Subjective cognitive complaints, *ND* Neurodegeneration, *FDG* ^18^F-Fluorodeoxyglucose, *MAC-Q* Memory assessment clinics questionnaire, *SMD* Subjective memory decline, *FTP* Flortaucipir, *SCD* Subjective cognitive decline, *CDR* Clinical dementia rating, *AD* Alzheimer’s disease, *SMI* Subjective memory impairment, *CMRglc* Cerebral metabolic rates for glucose, *ApoE Apolipoprotein E*, *FCSRT* Free and cued selective reminding test, *BNT* Boston naming test, *VOSP* Visual object and space perception battery, *ToL* Tower of London test, *CSF* Cerebrospinal fluid, *IP* Isoprostane, *SUVR* Standardized uptake value ratio, *SCI* Subjective cognitive impairment, *MCI* Mild cognitive impairment, *NC* Normal control, *PET* Positron emission tomography, *PiB* Pittsburgh compound B. ADNI: Alzheimer’s Disease Neuroimaging Initiative, *MRI* Magnetic resonance imaging, *MFQ* Mood and Feelings Questionnaire, *E-Cog* Everyday Cognition Scale, *RSFC* Resting-state functional connectivity

## Structural MRI and diffusion MRI

### Gray matter

Both cortical and subcortical atrophy develop as AD progresses, making its volumetry one of the most well-established imaging biomarkers of AD [62]. As preferential target locations of neurofibrillary tangles [45], the entorhinal cortex and hippocampus display marked atrophy in patients with dementia due to AD [63–66].

However, during the SCD stage, neuroimaging findings regarding gray matter changes remain mixed. Some studies have observed a decreased hippocampal volume in individuals with SCD both at baseline [59, 67–74] and during a significant longitudinal decline, with an annual decrease of 1.9% [75, 76], whereas other studies have not reported significant changes [77–83]. The heterogeneity of SCD may be one of the causes of the inconsistent results, as evidence has suggested that patients with clinically defined SCD who present with smaller brain volumes have a higher risk of developing dementia than community-recruited subjects with SCD [23]. When combined with genetic risk factors, SCD subjects who are *ApoE* ɛ4 carriers showed more severe atrophy in the left hippocampus [84] and an additive reduction in the right cortical surface area [85] than noncarriers; this outcome is consistent with the results from longitudinal studies [86]. Furthermore, some studies have investigated the volumetric differences in subcortical regions, including the cholinergic basal forebrain nuclei and the hippocampal subfield, between individuals with SCD and HCs. The findings have converged to suggest that SCD is associated with a significant reduction in the volume of the cholinergic basal forebrain and the CA1 region of the hippocampus compared with HCs [87–90]. In addition to a reduced subcortical volume, a thinner cortex, particularly in the temporal-parietal lobe, was associated with a more rapid memory deterioration and an increased risk of disease progression in subjects with SCD compared with HCs [91–94].

Interestingly, Peter et al. [95] used a multivariate pattern analysis (MVPA) to summarize the structural imaging profile of a subject into a single meaningful value via a multivariate classification framework. The researchers trained a classifier to separate individuals with dementia due to AD from HCs and found that the gray matter atrophy pattern of a subject with SCD was similar to the brain of a patient with dementia due to AD but was significantly different from an HC. The voxels with greatest contributions to the classification were mainly distributed in hippocampal and parahippocampal areas. The findings of the study by Peter and colleagues suggested that the multivariate analysis may represent a powerful method for detecting subtle and distributed changes in the early stages of AD.

Using brain network modeling methods, a gray matter network can be constructed by calculating the structural covariance between pairs of regions. According to previous studies, gray matter networks of patients with dementia due to AD tend to be more randomly organized with lower clustering coefficients and altered small-world properties [96–98], suggesting that AD may be a disconnection syndrome. Recently, Verfaillie et al. [99] and Tijms et al. [100] have reported altered patterns of gray matter networks in individuals with SCD that are similar to patients with dementia due to AD. Both groups of researchers showed that the gray matter network of individuals with SCD was more randomly organized than HCs, and the disrupted network properties were associated with a steeper decline in global cognition and a higher risk of disease progression. Moreover, Ten Kate et al. [101] observed an association between a higher level of global amyloid deposition and lower clustering and fewer small-world properties of gray matter structural networks in subjects with SCD. Overall, although some negative results have been reported, individuals with SCD related to AD have been repeatedly shown to present a reduced gray matter volume and cortical thickness and a disrupted gray matter network.

### White matter

Diffusion tensor imaging (DTI) has been increasingly applied to investigate microstructural alterations in the WM of patients with a neurodegenerative disease [102], which might reflect the pathological alterations of WM degeneration, such as axon loss, damage or demyelination. For patients with MCI and dementia due to AD, widespread disruptions with decreased fractional anisotropy (FA) and increased mean diffusivity (MD), particularly in the cingulum bundles and corpus callosum, and significant topological alterations of the brain structural connectome have been consistently reported [103–108].

In individuals with SCD, significantly decreased FA and increased MD have been observed in the cerebrum, mainly in the hippocampal body, entorhinal cortex and parahippocampal gyrus, uncinated fasciculi, longitudinal fasciculi and corpus callosum [77, 78, 109–113], whereas Kiuchi et al. and Viviano et al. reported no statistically significant differences in diffusion metrics [114, 115]. Various reasons for the discrepancy are plausible, including different operational definitions of SCD and differences in other factors (e.g., medication noncompliance, blood pressure, and scanner differences). Interestingly, by performing a whole-brain voxelwise analysis, Selnes et al. [116] found increased radial diffusivity (RD) and MD in widespread WM tracts but no changes in FA in subjects with SCD. This finding may indicate that FA is less sensitive than diffusivity metrics in revealing early pathological processes. In addition, genetic risk factors may aggravate degeneration in individuals with SCD. For instance, compared with noncarriers, *ApoE* ɛ4 carriers in SCD populations showed lower FA in the splenium of the corpus callosum and the anterior corona radiata [117]. Another investigation categorized individuals with SCD into a high-risk group and a low-risk group based on age, *ApoE* genotype, K-MMSE recall score and the Seoul Verbal Learning Test. The high-risk group showed more severe microstructural disruptions with reduced FA in the tracts connecting the hippocampus, parahippocampal gyrus, supramarginal gyrus and parts of the frontotemporal lobes [118].

In contrast to the quantification of local diffusion metrics, other scholars have investigated the topological organization of structural networks underlying SCD with a graph theory analysis. As shown in the study by Shu and colleagues, the SCD connectome exhibited lower global and local efficiency and reduced rich-club and local connections, which were correlated with impaired memory performance in subjects with SCD [119]. According to Yan et al., only a limited number of peripheral regions and the connectivity among nonhub regions were disrupted in patients with SCD, whereas rich-club integration remained stable in the early stage of SCD but subsequently progressed to exhibit disruptions associated with MCI and dementia due to AD [120]. Overall, topological measures of the brain structural connectome are sensitive metric of the early stage of AD, which establishes them as potential imaging markers of SCD.

The relationship between gray matter and WM alterations in subjects with SCD remains largely unknown. Research combining MRI volumetric and DTI measures suggested that individuals with SCD showed a lower entorhinal cortical volume, lower FA and higher MD in the hippocampal body and entorhinal WM [78]. According to another study [118], individuals with SCD with a high risk of progression had more severe WM disruption than the low-risk SCD group but no evident gray matter atrophy. Thus, relatively high-risk SCD may predict an underlying microstructural disruption in the WM that precedes gray matter atrophy during AD progression. However, during the SCD stage, the order of gray matter or WM alterations and the relationship between them require further investigation and elucidation to help us obtain a better understanding of the pathological mechanisms underlying AD development. The combination of multiple MRI modalities provides the opportunity to characterize biomarker features at different disease stages and precisely track the progression of neurodegenerative alterations (Table 2).

**Table 2:** Summary of structural and diffusion MRI studies

Authors	Definition of SCD	Modality	Design	Sample (mean age ± SD)	Main findings
Jessen et al. (2006) [81]	Memory clinic consultation for < 5 y SCD	T1 MRI	Cross-sectional	NC: *n* = 14 (66.5 ± 6.4) SCD: *n* = 12 (66.1 ± 7.3) MCI: *n* = 15 (68.2 ± 5.5) AD: *n* = 13 (68.8 ± 9.7)	Atrophy in entorhinal cortex not in hippocampus.
Saykin et al. (2006) [79]	Consensus evaluation using a composite index (multiple self and informant-based questionnaires)	T1 MRI	Cross-sectional	NC: *n* = 40 (71.0 ± 5.1) SCD: *n* = 40 (73.3 ± 6.0) MCI: *n* = 40 (72.9 ± 7.1)	Decreased gray matter in the MTL, frontotemporal and other neocortical regions in SCD and MCI. reduced hippocampal volumes only in MCI.
Nunes et al. (2010) [75]	Memory clinic consultation	T1 MRI	Longitudinal (NC: 3.4 years SCD: 3.4 years MCI: 3.7 years)	NC: *n* = 11 (69.5 ± 5.5) SCD: *n* = 15 (65.9 ± 7.7) MCI: *n* = 17 (70.8 ± 6.4)	SCD had decreased hippocampal volume longitudinal. MCI had decrease both in total hippocampal and amygdala volumes.
Shen et al. (2010) [80]	Consensus evaluation using a composite index (multiple self and informant-based questionnaires)	T1 MRI	Cross-sectional	NC: *n* = 38 (70.6 ± 5.2) SCD: *n* = 39 (72.8 ± 6.1) MCI: *n* = 37 (72.7 ± 7.1) AD: *n* = 11 (75.6 ± 6.8)	Both MCI group and the AD dementia group showed hippocampal volume reduction compared to NC and SCD.
Striepens et al. (2010)	Memory clinic consultation for <10 y SCD, informant confirmed	T1 MRI	Cross-sectional	NC: *n* = 48 (66.3 ± 6.2) SCD: *n* = 21 (65 ± 7.2)	The SCD had reduced volume of bilateral hippocampus, the bilateral entorhinal cortex and the right amygdala compared to the NC.
Stewart et al. (2011) [86]	2 binary questions (SCD when both positive)	T1 MRI	Longitudinal (4 years)	Baseline SCD: *n* = 1793 (72.4 ± 4.1) Follow-up SCD: *n* = 1336 (72.0 ± 4.0)	SCD at baseline was associated with subsequent change in hippocampal volume and at follow-up impairment was associated with previous change in hippocampus, CSF and gray matter volume.
Striepens et al. (2011) [84]	Memory clinic consultation for <10 y SCD, informant confirmed	T1 MRI	Cross-sectional	NC *ApoE* ɛ4+: *n* = 16 (65.9 ± 7.2) NC *ApoE* ɛ4-: *n* = 56 (67.4 ± 7.7) SCD *ApoE* ɛ4+: *n* = 11 (66.8 ± 6.8) SCD *ApoE* ɛ4-: *n* = 30 (68.5 ± 7.2)	*ApoE* ɛ4 carriers with SMI performed worse on the episodic memory and showed smaller left hippocampal volumes. The *ApoE* ɛ4 carriers without SMI performed better on episodic memory and had larger right hippocampal volumes.
Scheef et al. (2012) [59]	Memory clinic consultation for <10 y SCD with worry, informant confirmed	T1 MRI;	Longitudinal (NC: 34.6 months SCD: 35.5 months)	Baseline NC: *n* = 56 (66.4 ± 7.2) SCD: *n* = 31 (67.6 ± 6.2) Follow-up NC: *n* = 48 (66.5 ± 7.2) SCD: *n* = 27 (67.4 ± 6.5)	SCD had reduced gray matter volume in the right hippocampus.
Kim et al. (2013) [68]	Reason for seeking help: memory or health promotion?	T1 MRI	Cross-sectional	NC: *n* = 28 (70.7 ± 5.5) SCD: *n* = 90 (65.8 ± 8.5)	The SCD showed significantly smaller hippocampal and amygdala volumes. Association between lower GDS score and smaller hippocampal volume SCD, and association between higher GDS score and smaller amygdala volume NC.
Peter et al. (2014) [95]	Memory clinic consultation for <10 y SCD with worry, informant confirmed	T1 MRI	Cross-sectional	NC: *n* = 53 (67.1 ± 6.1) SCD: *n* = 24 (66.0 ± 7.1)	SCD showed greater similarity to a dementia gray matter pattern compared with NC. Association between episodic memory decline and a dementia gray matter pattern in SCD.
Cherbuin et al. (2015) [76]	1 binary question	T1 MRI	Longitudinal (4 years)	NC: *n* = 218 (62.7 ± 1.32) W1 SCD: *n* = 70 (62.1 ± 1.4) W2 SCD: *n* = 56 (62.4 ± 1.5) W1 + W2 SCD: *n* = 39 (62.3 ± 1.4)	SCD at baseline was not associated with hippocampal atrophy. SCD at follow-up was associated with greater hippocampal atrophy.
Meiberth et al. (2015) [91]	Memory clinic consultation for <10 y SCD, informant confirmed	T1 MRI	Cross-sectional	NC: *n* = 69 (66.1 ± 6.9) SCD: *n* = 41 (68.9 ± 7.2)	SCD showed thickness reduction in left entorhinal cortex compared to NC.
Perrotin et al. (2015) [88]	Memory clinic consultation	T1 MRI	Cross-sectional	NC: *n* = 40 (69.4 ± 6.4) SCD: *n* = 17 (69.1 ± 8.5) AD: *n* = 21 (68.3 ± 9.5)	SCD showed TIV-normalized volume decrease in hippocampus compared to NC.
Schultz et al. (2015) [92]	1 binary question	T1 MRI	Cross-sectional	SCD: *n* = 77 (54.3 ± 6.1) NC: *n* =184 (54.4 ± 6.4)	SCD showed cortical thinning in the entorhinal, fusiform, posterior cingulate, and inferior parietal cortices and reduced amygdala volume compared with NC
Cantero et al. (2016) [87]	Questionnaire, structured interview	T1 MRI	Cross-sectional	NC: *n* = 47 (68.1 ± 3.2) SCD: *n* = 48 (69.6 ± 4.3)	SCD showed decreased volumes of CA1, CA4, dentate gyrus and molecular layer compared to NC. Lower volume of the dentate gyres associates with poorer memory performance.
Hong et al. (2016) [118]	Memory clinic consultation	T1 MRI DTI	Cross-sectional	Low risk: *n* = 27 (62.1 ± 7.1) High risk: *n* = 19 (67.1 ± 6.5)	The high-risk group showed lower FA in the hippocampus, parahippocampal gyrus, supramaiginal gyrus and parts of fronto-temporal lobes, but no gray matter atrophy.
Jung et al. (2016) [121]	Memory clinic consultation	T1 MRI	Cross-sectional	SMI: n=612(64.9 ± 6.9)	Individuals with different subtype atrophy showed difference in age, gender, vascular risk factors and depression. Combination of these factors classified the temporal atrophy subtype and the minimal atrophy subtype with 73.2% and 76.0% accuracy.
Lee et al. (2016) [117]	Memory clinic consultation	T1 MRI DTI	Cross-sectional	*ApoE* ɛ4+: *n* = 13 (66.4 ± 6.3) *ApoE* ɛ4-: *n* = 13 (66.2 ± 7.8)	*ApoE* ɛ4+ SCD showed gray matter atrophy and lower FA compared with *ApoE* ɛ4- SCD.
Rogne et al. (2016) [69]	1 binary question	T1 MRI	Cross-sectional	NC: *n* = 58 (70.6 ± 6.7) SCD: *n* = 25 (70.0 ± 9.1) MCI: *n* = 115 (74.5 ± 7.5)	SCD had larger lateral ventricles and smaller hippocampal volumes than NC.
Sun et al. (2016) [122]	Memory clinic consultation	T1 MRI rs-fMRI	Cross-sectional	NC: *n* = 61 (64.1 ± 8.6) SCD: *n* = 25 (65.5 ± 6.1)	SCD showed higher ALFF but no differences in gray matter volume
Verfaillie et al. (2016) [93]	Memory clinic consultation	T1 MRI	Cross-sectional	SCD stable: *n* = 253 (61 ± 9) SCD progression: *n* = 49 (69 ± 6)	Hippocampal volumes, thinner cortex of the AD-signature and various AD-signature subcomponents were associated with increased risk of clinical progression
Lauriola et al. (2017) [123]	Subjective cognitive decline Questionnaire	T1 MRI	Cross-sectional	NC: *n* = 38 (64.0 ± 5.1) SCD: *n* = 32 (64.8 ± 6.3)	SCD showed increased nighttime wakefulness and reduced sleep efficiency.
Norton et al. (2017) [124]	Memory Complaint Scald in Spanish	T1 MRI	Cross-sectional	Noncarriers: *n* = 26 (37.2 ± 6.5) Carriers: *n* = 26 (35.6 ± 7.7)	PSEN-1 E280A mutation carrier showed decreased hippocampal volume in SCD compared to noncarriers.
Perrotin et al. (2017) [23]	Memory clinic consultation	^18^F-florbetapir PET and T1 MRI	Cross-sectional	NC: *n* = 35 (65.8 ± 8.6) SCD community: *n* = 35 (70.8 ± 7.5) SCD clinic: n 28 (67.6 ± 7.7)	SCD showed increased amyloid deposition. Subclinical depression and hippocampal atrophy were associated with medical help seeking.
Risacher et al. (2017) [125]	Cognitive change Index	^18^F-florbetapir and T1 MRI	Cross-sectional	NC: *n* = 19 (68.5 ± 6.9) SCD: *n* = 10 (72.2 ± 6.4) MCI: *n* = 5 (75.7 ± 10.6)	Lower UPSIT scores were associated with increased temporal, parietal tau burden and temporal lobe atrophy in the full sample and in NC and SCD only.
Hafkemeijer et al. (2013) [73]	Memory clinic consultation	T1 MRI	Cross-sectional	NC = 29 (71.3 ± 3.6) SCD: *n* = 25 (71.4 ± 9.2)	Reduced gray matter volume in DMN regions.
Platero et al., (2019) [82]	SCD-I Working Group	T1 MRI	Cross-sectional	NC: *n* = 70 (70.3 ± 4.5) SCD: *n* = 87 (71.7 ± 5.1) MCI: *n* = 137 (73.9 ± 5.0) AD: *n* = 13 (75.6 ± 5.0)	No differences in hippocampal volumes between NC and SCD.
Sanchez-Benavid et al., (2018) [72]	1 binary question and SCD-Q questionnaire	T1 MRI	Cross-sectional	NC: *n* = 2098 (55.41 ± 6.62) SCD-: *n* = 319 (55.62 ± 6.22) SCD+: *n* = 253 (59.10 ± 7.12)	SCD+ subjects showed lower gray matter volumes.
Sun et al., (2019) [85]	Memory clinic consultation for < 5 y SCD	T1 MRI	Cross-sectional	NC: *n* = 73 (64.55 ± 5.52) SCD: *n* = 65 (65.85 ± 4.85)	Decreased total cortical volumes and cortical surface area in SCD. SCD *ApoE* ɛ4 carriers showed additive reduction in the right cortical surface area.
Tepest et al., (2018) [83]	Memory clinic consultation	T1 MRI	Cross-sectional	NC: *n* = 13 (67.5 ± 5.5) SCD: *n* = 14 (66.4 ± 7.3) MCI: *n* = 15 (68.2 ± 5.4) AD: *n* = 12 (69.2 ± 10.0)	No differences in hippocampal surface between SCD and NC.
Tijms et al., (2018) [100]	Memory clinic consultation	T1 MRI	Cross-sectional	sSCD: *n* = 100 (67 ± 8) pSCD : *n* = 122 (68 ± 8)	Lower network parameter values related with increased risk for progression.
Rooden et al., (2018) [74]	Memory clinic consultation	T1 MRI	Cross-sectional	NC: *n* = 42 (68 ± 9.2) SCD: *n* = 25 (68 ± 9.1)	SCD showed hippocampal atrophy.
Zhao et al., (2019) [89, 126]	SCD-I Working Group	T1 MRI	Cross-sectional	NC: *n* = 42 (64.24 ± 6.16) SCD: *n* = 35 (64.53 ± 7.29) aMCI: *n* = 43 (67.47 ± 10.03) AD: *n* = 41 (68.88 ± 7.86)	No difference in hippocampal volume between NC and SCD.
Ryu et al. (2017) [78]	Memory clinic consultation	T1 MRI and DTI	Cross-sectional	NC: *n* = 27 (70.6 ± 6.1) SCD: *n* = 18 (69.9 ± 6.3)	SCD showed lower entorhinal cortical volumes and lower FA and higher MD in the hippocampal body and entorhinal WM compared with NC.
Fan et al. (2018) [77]	Memory clinic consultation	T1 MRI and DTI	Cross-sectional	NC: *n* = 34 (67.8 ± 7.4) SCD: *n* = 43 (66.1 ± 7.0) aMCI: *n* = 44 (73.9 ± 8.0)	SCD showed cortical atrophy and decreased mean FA.
Niemantsverdriet et al. (2018) [127]	Criteria by SCD-I	T1 MRI	Cross-sectional	NC: *n* = 93 (67.3 ± 8.5) SCD: *n* = 102 (68.6 ± 9.8) MCI: *n* = 379 (74.6 ± 8.0) AD: *n* = 313 (77.5 ± 8.0)	Baseline whole brain, gray matter, cortical gray matter and increased CSF volumes predicted cognitive impairment
Verfaillie et al. (2018) [94, 99, 128]	Referred by general practitioners or medical specialists	T1 MRI	Cross-sectional	SCD: *n* = 233 (52.8 ± 9.2)	SCD with faster subsequent memory loss was associated with thinner cortex of the frontal and occipital cortices.
Verfaillie et al. (2018) [94, 99, 128]	Memory clinic consultation	T1 MRI	Cross-sectional	SCD: *n* = 231 (63.0 ± 9.2)	SCD with lower network size was associated with steeper decline in language.
Yue et al. (2018) [71]	1 binary questions	T1 MRI	Cross-sectional	NC: *n* = 67 (67.7 ± 6.6) SCD: *n* =111 (69.8 ± 7.6) MCI: *n* = 30 (75.5 ± 7.6)	The SCD showed decreased right hippocampal and amygdala volume than NC. Right hippocampal and amygdala volume was correlated to MMSE and MoCA in SCD.
Lee et al. (2016) [117]	Memory clinic consultation	T1 MRI DTI	Cross-sectional	*ApoE* ɛ4+: *n* = 13 (66.4 ± 6.3) *ApoE* ɛ4-: *n* = 13 (66.2 ± 7.8)	*ApoE* ɛ4+ SCD showed gray matter atrophy and lower FA compared with *ApoE* ɛ4- SCD.
Brueggen et al., (2019) [111]	Memory clinic consultation	T1 MRI, DTI	Cross-sectional	NC: *n* = 93 (68.5 ± 5.1) SCD: *n* = 98 (71.3 ± 5.9) MCI: *n* = 45 (72.3 ± 5.7) AD: *n* = 35 (73.5 ± 6.8)	SCD showed higher MD, lower MO and FA.
Kiuchi et al., (2014) [114]	Memory clinic consultation	T1 MRI, DTI	Cross-sectional	NC: *n* = 41 (75.2 ± 5.34) SCD: *n* = 28 (70.5 ± 7.30) MCI: *n* = 43 (74.6 ± 6.40) AD: *n* = 39 (73.2 ± 7.98)	No differences between NC and SCD.
Li et al., (2016) [109]	SCD-I Working Group	DTI	Cross-sectional	NC: *n* = 37 (65.1 ± 6.8) SCD: *n* = 27 (65.3 ± 8.0) aMCI: *n* = 35 (69.2 ± 8.6) AD: *n* = 25 (68.3 ± 9.4)	SCD showed decreased FA, increased MD and RD.
Ohlhauser et al., (2019) [112]	Cognitive Change Index test		Cross-sectional	NC: *n* = 44 (72.49 ± 6.37) SCD: *n* = 30 (72.94 ± 4.79)	SCD showed lower WM integrity and DTI metrics related with executive function in SCD.
Viviano et al., (2019) [115]	Memory clinic consultation		Cross-sectional	NC: *n* = 48 (66.96 ± 8.79) SCD: *n* = 35 (68.51 ± 7.66)	No differences in diffusion measures between SCD and NC
Yasuno et al., (2015) [113]	Memory clinic consultation		Cross-sectional	NC: *n* = 30 (72.2 ± 4.8) SCD: *n* = 23 (69.6 ± 8.0)	SCD showed reduced WM connections.
Selnes et al (2012) [116]	Memory clinic consultation	T1 MRI and DTI	Cross-sectional	NC: *n* = 21 (49 - 77) SCD: *n* = 16 (45 - 71) MCI: *n* = 50 (45 - 77)	SCD had higher DR and MD in posterior cingulate, retrosplenial and middle cortices.
Shu et al (2018) [119]	Memory clinic consultation	DTI	Cross-sectional	NC: *n* = 51 (62.2 ± 9.1) SCD: *n* = 36 (63.5 ± 8.7)	SCD had lower global and local efficiency and reduced rich-club and local connections which were correlated with the impaired memory performance.
Wang et al. (2012) [110]	Memory clinic consultation	DTI	Cross-sectional	NC: *n* = 35 (71.6 ± 5.2) SCD: *n* = 29 (73.4 ± 6.3) MCI: *n* = 28 (74.3 ± 5.8)	SCD had FA, DR, DA and MD values that were intermediate to the MCI and NC.
Yan et al. (2018) [120]	Memory clinic consultation	DTI	Cross-sectional	NC: *n* = 62 (63.3 ± 8.1) SCD: *n* = 47 (65.3 ± 8.4) aMCI: *n* = 60 (67.3 ± 9.4) d-AD: *n* = 55 (70.9 ± 9.8)	SCD showed disrupted peripheral regions and reduced connectivity similar to MCI and dementia due to AD. The rich club organization remain stable in the earliest stage only in SCD

*SCC* Subjective cognitive complaints, *ND* Neurodegeneration, *SMD* Subjective memory decline, *FTP* Flortaucipir, *SCD* Subjective cognitive decline, *CDR* Clinical dementia rating, *AD* Alzheimer’s disease, *SMI* Subjective memory impairment, *CMRglc* Cerebral metabolic rates for glucose, *ApoE Apolipoprotein E*, *FCSRT* Free and cued selective reminding test, *BNT* Boston naming test, *VOSP* Visual object and space perception battery, *ToL* Tower of London test, *CSF* Cerebrospinal fluid, *IP* Isoprostane, *SUVR* Standardized uptake value ratio, *SCI* Subjective cognitive impairment, *MCI* Mild cognitive impairment, *aMCI* Amnestic MCI, *NC* Normal control, *PET* Positron emission tomography, *ADNI* Alzheimer’s Disease Neuroimaging Initiative, *MRI* Magnetic resonance imaging, *DTI* Diffusion tensor imaging, *WM* White matter, *FA* Fractional anisotropy, *MD* Mean diffusivity, *RD* Radial diffusivity, *rs-fMRI* Resting-state functional MRI, *DMN* Default mode network

## Functional MRI

### Resting-state fMRI

Resting-state fMRI (rs-fMRI) is a noninvasive technique that detects alterations of spontaneous brain activity and interregional functional connectivity (FC) by measuring intrinsic blood oxygen level-dependent (BOLD) low-frequency signal fluctuation. Based on accumulating evidence, the FC of the default mode network (DMN) is selectively disrupted in patients with MCI and dementia due to AD [52, 129, 130]. The DMN can be detected in a resting state and plays an important role in episodic memory processing, self-reference, social cognition and overall brain function [131–133]. The DMN consists of a set of highly interconnected brain regions, including the posterior cingulate cortex, medial prefrontal cortex, lateral temporoparietal cortices and the hippocampus [134]. Importantly, these regions are among the earliest locations to show gray matter atrophy, hypometabolism and amyloid deposition during the initiation of AD pathology.

For subjects with SCD, our group reported a higher amplitude of low-frequency fluctuations (ALFF) of spontaneous brain activity in the left inferior parietal lobule and right middle occipital gyrus and a lower ALFF in the precuneus and cerebellum than in HCs [122, 135]. The alterations in ALFF were correlated with the verbal episodic memory scores of subjects with SCD [122]. The increased ALFF in SCD subjects may reflect a compensatory mechanism for functional deficits in the preclinical stage of AD. Yang et al. observed a higher accuracy of discriminating individuals with SCD from HCs when ALFF and fractional ALFF features were combined than when only single features were used [135].

Other studies have focused on the alterations in intrinsic functional networks. Subjects with SCD exhibited higher FC in the retrosplenial cortex and precuneus and lower FC in the frontal-parietal cortex and putative posterior memory system [115, 136, 137]. As an important potential biomarker, the DMN has also received increasing attention in individuals with SCD. However, the results remain heterogeneous. In terms of connectivity between the DMN and other regions, Verfaillie et al. [128] found increased connectivity between the posterior DMN and the medial temporal memory system in subjects with SCD; in contrast, decreased connectivity between the DMN and hippocampus was observed in individuals with SCD in another study [138]. Regarding the connectivity within the DMN, one study reported reduced DMN connectivity in individuals with SCD relative to HCs [139], conflicting with the findings of another study [73]. In addition, Yasuno and colleagues reported reduced FC in cortical midline structures where some of the DMN regions are located in individuals with SCD [113]. Furthermore, some studies also explored the interactions of SCD with other risk factors for dementia due to AD and their effects on FC. Cavedo et al. [60] found sex-specific alterations in the resting state (FC) of the DMN. The study conducted by Chiesa et al. revealed that *ApoE* ɛ4 carriers presented a slower longitudinal increase in FC in the frontal lobes than noncarriers [140]. Overall, these findings indicate an important role for the functional network, particularly the DMN, in SCD, suggesting that the DMN may represent a specific target for the early intervention and treatment of AD, although these results are heterogeneous and more studies based on rs-fMRI are needed to explore the role of the DMN in SCD.

### Task-based fMRI

Task-based fMRI has been widely used to explore changes in functional activity during different cognitive tasks by examining the changes in BOLD signals. Medial temporal lobe hypoactivation, parietal hyperactivation, and frontal hyperactivation during memory tasks have been reported in patients with MCI and dementia due to AD [141–144]. Task-based fMRI studies of subjects with SCD remain scarce, and the current findings are controversial.

In one study, subjects with SCD showed increased activation in the middle frontal gyrus, the precuneus and the cingulate gyrus relative to HCs during working memory tasks [145]. However, a different effect was observed in another study [146], which showed that SCD was associated with reduced activation in the right hippocampus and increased activation in the right dorsolateral prefrontal cortex during an episodic memory recall task. In addition, Rodda et al. reported increased activation in the prefrontal cortex, left medial temporal lobe, bilateral thalamus, posterior cingulate and caudate of individuals with SCD during the encoding of novel words and a divided attention task [147, 148]. Notably, these regions displaying greater activation during different tasks were proposed to be mainly involved in the DMN, which may reflect the deployment of some compensatory processes. However, researchers have not yet clearly determined whether these differences are specific to successful memory encoding or related to general cognitive processes. Hayes and colleagues applied a subsequent memory paradigm to examine potential differences in the subsequent memory effect between cognitively intact older adults with and without SCD [149]. The researchers found that SCD was associated with more limited subsequent memory effects on the occipital lobe, superior parietal lobe, and posterior cingulate cortex and more disrupted subsequent memory effects on areas of the DMN. Interestingly, Hu and colleagues identified an association between increased delay discount in individuals with SCD and changes in the brain network related to episodic memory [150]. Hence, the altered functional activation patterns observed during memory tasks may indicate brain functional reorganization due to SCD. However, some advanced modeling approaches, such as dynamic causal modeling, should be productively applied to investigate early functional alterations in individuals with SCD.

### Arterial spin labeling

Arterial spin labeling (ASL) MRI is a noninvasive technique for quantifying cerebral perfusion that has been implicated as a useful biomarker of the early stages of AD [151]. Patients with MCI exhibit hypoperfusion in the temporal parietal cortex [152–155], and patients with dementia due to AD present with decreased cerebral blood flow (CBF) in a wide range of brain areas [152, 156–159] compared with cognitively normal adults. In addition, some studies even reported that cognitively normal adults with the *ApoE* ɛ4 allele [154, 160, 161] and a maternal family history of AD [152] had an altered CBF (including a greater decrease in CBF over time, reduced CBF and increased resting-state CBF) compared with controls.

However, fewer ASL-MRI studies of individuals with SCD are available. To the best of our knowledge, only two studies have explored the differences in CBF between individuals with SCD and HCs. According to Hays et al., patients with SCD exhibited lower CBF in the medial orbitofrontal cortex and higher CBF in the right putamen than HCs [162], while de Eulate et al. did not observe any differences in total blood flow between individuals with SCD and HCs [163].

The results for the relationship between cognition and CBF are also inconsistent. Hays et al. observed negative association between verbal memory and CBF within the posterior cingulate cortex, middle temporal gyrus, hippocampus, fusiform gyrus and inferior frontal gyrus in patients with SCD [162], while Leeuwis et al. did not observed correlation between CBF and cognition in patients with SCD [164].

These controversial results may be due to the use of different cohorts and image processing methodologies. Hays et al. used voxelwise statistics among community-dwelling older volunteers, and the other two studies focused on defined ROI regions in the Amsterdam Dementia Cohort. In summary, ASL-MRI has vast potential as a biomarker of SCD, but additional studies using this modality are needed (Table 3).

**Table 3:** Summary of functional MRI studies

Authors	Definition of SCD	Modality	Design	Sample (mean age ± SD)	Main findings
Dummas et al. (2013) [145]	Endorsed more than 20% of the items on the complaint inventory	Task-fMRI	Cross-sectional	NC: *n* = 11 (56.8 ± 1.9) SCD: *n* = 12 (57.1 ± 2.3)	SCD had increased activations in middle frontal gyrus, precuneus and cingulate gyrus compared to NC.
Erk et al. (2011) [146]	Memory clinic consultation	Task-fMRI	Cross-sectional	NC: *n* = 20 (66.8 ± 5.4) SCD: *n* = 19 (68.4 ± 5.7)	SCD was associated with a reduction in right hippocampal activation during episodic memory recall in the absence of performance deficits and increased activation of the right dorsolateral prefrontal cortex.
Rodda et al. (2009) [147]	Self-perceived memory difficulties persistent and severe enough to seek advice despite normal cognition	Task-fMRI	Cross-sectional	NC: *n* = 10 (68.0 ± 13.5) SCD: *n* = 10 (64.2 ± 5.6)	SCD exhibited increased activation in left during the divided attention task.
Hu et al. (2017) [150]	Criteria by SCD-I	Task-fMRI	Cross-sectional	NC: *n* = 24 (66.5 ± 7.2) SCD: *n* = 20 (68.3 ± 7.9)	Subtle neuronal network disruptions in SCD.
Hayes et al. (2017) [149]	Worrisome decline in memory	Task-fMRI	Cross-sectional	NC: *n* = 41 (67.5 ± 9.1) SCD: *n* = 23 (68.6 ± 8.2)	SCD showed a more negative subsequent memory effects in the default mode network.
Dillen et al. (2017) [138]	A cut-off value of≥25 on the memory complaint questionnaire but average scores on neuropsychological tests	rs-fMRI	Cross-sectional	NC: *n* = 25 (62.4 ± 7.0) SCD: *n* = 28 (65.8 ± 7.8) Prodromal AD: *n* = 25 (70.8 ± 6.2)	SCD showed decreased connectivity between DMN and hippocampus.
Hafkemeijer et al. (2013) [73]	Memory complaints but normal cognition	T1 MRI and rs-fMRI	Cross-sectional	NC: *n* = 29 (71.3 ± 3.4) SCD: *n* = 25 (71.4 ± 9.2)	SMC showed increased FC in the default mode network.
Sun et al. (2016) [122]	Self-reported persistent decline in memory compared with a previous state but normal cognition	T1 MRI and rs-fMRI	Cross-sectional	NC: *n* = 61 (64.1 ± 8.6) SCD: *n* = 25 (65.5 ± 6.1)	SCD had higher ALFF values in the left inferior parietal lobule and right middle occipital gyrus than control subjects, which were correlated with verbal episodic memory scores.
Verfaillie et al. (2018) [128]	1 binary question	rs-fMRI	Longitudinal (one year)	Baseline NC: *n* = 56 (64 ± 5) Baseline SCD: *n* = 68 (64 ± 5) Follow-up NC: *n* = 29 (65 ± 6) Follow-up SCD: *n* = 30 (65 ± 6)	SCD showed increased pDMN–MTMS connectivity. Higher connectivity between MTMS and the rest of brain was associated with better baseline immediate memory, attention, and global cognition. Higher MTMS and pDMN–MTMS connectivity were associated with lower immediate memory over time.
Wang et al. (2013) [139]	Endorsed more than 20% of the items on the Cognitive Complaint Index	rs-fMRI	Cross-sectional	NC: *n* = 16 (70.7 ± 6.0) SCD: *n* = 23 (70.1 ± 7.3) MCI: *n* = 18 (73.7 ± 9.1)	SCD showed decreased DMN connectivity in the right hippocampus compared to NC and higher connectivity compared to MCI.
Yasuno et al. (2015) [113]	Reisberg criteria	rs-fMRI	Cross-sectional	NC: *n* = 30 (72.2 ± 4.8) SCD: *n* = 23 (69.6 ± 8.0)	SCD showed reduced FC in cortical midline structures
Cavedo et al. (2018)	subjective memory complaints	^18^F-florbetapir-PET FDG-PET MRI	Cross-sectional	Women: n=201 (76.02±3.24) Men: *n* = 117(76.05±3.85)	Men had lower resting-state FC.
Chiesa et al., (2019) [140]	2 binary questions	rs-fMRI	Cross-sectional	*ApoE* ɛ4+: 44 (75.6 ± 3.5) *ApoE* ɛ4-: 180 (75.5 ± 3.4)	*ApoE* ɛ4+ showed slower increase in FC in frontal lobes.
Dillen et al., (2016) [136]	Structural questionnaire	rs-fMRI	Cross-sectional	NC: *n* = 25 (62.4 ± 7.0) SCD: *n* = 27 (65.7 ± 7.9) AD: *n* = 24 (71.0 ± 6.2)	Higher FC from RSC to frontal cortex in SCD.
Dong et al., (2018) [137]	Memory clinic consultation	rs-fMRI	Cross-sectional	NC: *n* = 39 (82.89 ± 4.13) SCD: *n* = 39 (83 ± 4.43)	Lower aFCS in SCD.
Viviano et al., (2019) [115]	2 binary questions	rs-fMRI	Cross-sectional	NC: *n* = 48 (66.96 ± 8.79) SCD: *n* = 35 (68.51 ± 7.66)	SCD showed lower average FC.
Eulate el al., (2017) [163]	Memory clinic consultation	ASL	Cross-sectional	NC: *n* = 32 (72.3 ± 5.6) SCD: *n* = 28 (67.3 ± 7.8) MCI: *n* = 34 (73.7 ± 7.5) AD: *n* = 21 (75.8 ± 6.2)	No differences in CBF between SCD and HC.
Hays et al., (2018) [162]	Memory clinic consultation	ASL	Cross-sectional	NC: *n* = 35 (73 ± 6.25) SCD: *n* = 35 (72.54 ± 5.07)	SCD showed negative associations between verbal memory and CBF.
Leeuwis et a., (2017) [164]	Memory clinic consultation	ASL	Cross-sectional	SCD: *n* = 143 (56.69 ± 8.69) MCI: *n* = 95 (65.24 ± 7.28) AD: *n* = 161 (65.93 ± 7.04)	No correlation between CBF and cognition.
Yang et al., (2019) [165]	SCD-I Working Group	rs-fMRI	Cross-sectional	NC: *n* = 55 (63.41 ± 7.97) SCD: *n* = 43 (65.09 ± 8.66) aMCI: *n* = 52 (68.06 ± 9.32) AD: *n* = 44 (70.98 ± 10.02)	SCD showed lower fALFF.

*FDG* ^18^F-Fluorodeoxyglucose, *SCD* Subjective cognitive decline, *AD* Alzheimer’s disease, *MCI* Mild cognitive impairment, *aMCI* Amnestic MCI, *NC* Normal control, *PET* Positron emission tomography, *MRI* Magnetic resonance imaging, *fMRI* Functional MRI, *rs-fMRI* Resting-state fMRI, *MTL* Medial temporal lobe, *ALFF* Amplitude of low-frequency fluctuations, *fALFF* Fractional ALFF, *DMN* Default mode network, *pDMN* Posterior DMN. SMC: Subjective memory complaints, *MTMs* Medial temporal memory system, *RSN* Resting-state networks, *VIS* Visual, *CCI* Cognitive complaint index, *ASL* Arterial spin labeling, *CBF* Cerebral blood flow, *SCD-I* Subjective Cognitive Decline Initiative, *FC* Functional connectivity, *ApoE Apolipoprotein E*

## EEG/MEG

Electroencephalography (EEG) and magnetoencephalography (MEG) are noninvasive techniques that record the electrical activity and magnetic fields generated by neuronal activity in the brain, respectively. During the last few decades, many studies have investigated the alterations in EEG and/or MEG signals in patients with MCI and dementia due to AD and have reported slowing brain rhythms and abnormal FC in the patients [166–169].

While the advanced stages of AD may be associated with functional disconnection [170], earlier stages may be apparent in terms of spectral measures and cortical rhythms detected using EEG [171–173]. Indeed, spectral data have shown a higher alpha power in patients with SCD that was most strongly correlated with a decline in verbal memory performance and the working memory reaction time [171]. In addition, Gouw et al. [173] reported association between abnormal delta, theta and alpha power and alpha peak frequency with clinical progression. In addition, amplitude abnormalities in delta, theta, and alpha rhythms have been recorded for individuals with SCD compared with HCs, suggesting that individuals with SCD present an abnormal pattern of dominant cortical alpha rhythms [172]. Furthermore, when separating individuals with SCD into decliners and nondecliners based on whether cognition decreased longitudinally, decliners showed increases in theta power, slowing of the mean frequency and changes in covariance among regions, particularly in the right hemisphere [174].

Using MEG, researchers have observed a significant alteration in spontaneous alpha activity in elderly participants with SCD, and this alteration was related to a decrease in cognitive performance [175]. An increase in brain activation in subjects with SCD and MCI during a memory task has also been reported [176]. Then, based on connectivity-based analyses, researchers revealed that participants with MCI and SCD exhibited a very similar pattern of alterations combining hypersynchronization over anterior brain regions (affecting the connection between the cingulate gyrus, frontal regions and anterior temporal areas) and hyposynchronization affecting more posterior areas (including parietal and medial temporal structures and occipital regions) [168]. Furthermore, subjects with SCD showed decreased clustering and transitivity in theta and beta bands, but increased modularity and transitivity in the alpha band, based on a graph theory analysis [177].

Overall, the aforementioned evidence supports the hypothesis that EEG/MEG measures play important roles in detecting early functional brain alterations in individuals with SCD and may serve as early imaging biomarkers of AD initiation (Table 4).

**Table 4:** Summary of EEG and MEG studies

Authors	Definition of SCD	Modality	Design	Sample (mean age ± SD)	Main findings
Alexander et al. (2006) [171]	Memory clinic consultation	EEG	Cross-sectional	NC: *n* = 79 (63.1 ± 7.9) SCD: *n* = 100 (64.9 ± 8.7)	SCD showed higher alpha power and changes in wave activity both related to decreased memory.
Babiloni et al. (2010) [172]	Memory clinic consultation	EEG	Cross-sectional	NC: *n* = 79 (69.7 ± 0.9) SCD: *n* = 53 (69.0 ± 1.0) aMCI: *n* = 92 (72.0 ±) naMCI: *n* = 51 (73.0 ± 1.1)	SCD showed greater frontal delta sources and lower parietal and occipital theta sources in amplitude.
Gouw et al. (2017) [173]	Criteria by SCD-I	EEG	Cross-sectional	SCD: *n* = 63 (66.2 ± 8.2) MCI: *n* = 142 (68.3 ± 7.4)	In SCD, higher delta and theta power and lower alpha power and peak frequency were associated with clinical progression
Teipel et al. (2018) [178]	2 binary questions	EEG and ^18^F-florbetapir-PET	Cross-sectional	SCD amyloid-: *n* = 255 (75.9 ± 3.5) SCD amyloid +: *n* = 63 (76.7 ± 3.5)	Amyloid accumulation does not impair cortical FC in SCD.
Lopez-Sanz et al. (2016) [175]	Self-reported cognitive concerns, older than 60	MEG and T1 MRI	Cross-sectional	NC: *n* = 39 (70.4 ± 3.7) SCD: *n* = 41 (71.6 ± 4.5) MCI: *n* = 51 (73 ± 3.7)	SCD and MCI exhibited a similar reduction in alpha band activity compared with NC. MCI showed a slowing in alpha peak frequency compared with both SCD and NC.
Lopez-Sanz et al. (2017) [168]	Self-reported cognitive concerns, older than 60	MEG and T1 MRI	Cross-sectional	NC: *n* = 39 (70.4 ± 3.7) SCD: *n* = 41 (71.6 ± 4.5) MCI: *n* = 51 (73 ± 3.7)	SCD and MCI showed lower FC in a hyper-synchronized anterior network and a posterior network.
Lopez-Sanz et al. (2017) [168]	Self-reported cognitive concerns, older than 60	MEG and T1 MRI	Cross-sectional	NC: *n* = 63 (70.7 ± 4.5) SCD: *n* = 55 (71.0 ± 5.0) MCI: *n* = 69 (71.9 ± 4.2)	SCD showed decreased clustering and transitivity in theta and beta bands but increased modularity and transitivity in alpha band.
Maestu et al. (2011) [176]	Patient stating that their memory function has deteriorated compared to earlier stages in life	MEG	Cross-sectional	NC: *n* = 6 (72 ± 8) SCD: *n* = 12 (72 ± 6) MCI: *n* = 21 (75 ± 3)	The SCD showed higher activation than the control group in posterior ventral regions and in the dorsal pathway. MCI patients showed higher activation than the control group in the posterior part of the ventral pathway.
Prichep et al., (2006) [174]	Memory clinic consultation	EEG	Cross-sectional	Nondecliners: *n* = 17 (70.0 ± 4.1) Decliners: *n* =27 (73.5 ± 4.9)	Decliners showed increase in theta power.

*SCD* Subjective cognitive decline, *AD* Alzheimer’s disease, *MCI* Mild cognitive impairment, *aMCI* Amnestic MCI, *naMCI* Non-amnestic MCI, *NC* Normal control, *PET* Positron emission tomography, *MRI* Magnetic resonance imaging, *EEG* Electroencephalography, *MEG* Magnetoencephalography, *SCD-I* Subjective Cognitive Decline Initiative, *FC* Functional connectivity

## Multimodal neuroimaging studies

Multimodal neuroimaging techniques combing PET and MRI have been used with increasing frequency to improve our understanding of the pathological interactions underlying SCD due to AD [179]. Abnormal amyloid pathology is earliest pathological change and triggers downstream neurodegeneration events [2]. A between-group analysis performed by Chetelat et al. indicated that, in participants with SCD, individuals with a higher level of amyloid deposition showed significant gray matter atrophy compared with individuals with a low level of amyloid deposition [180]. Further correlation analyses between imaging modalities also supported the relationship between amyloid pathology and reduced integrity of brain structures in both the gray matter and WM ranging from voxel level to brain connectome properties in subjects with SCD [101, 181, 182]. Ferreira et al. tested a disease severity index generated from a multivariate analysis involving amyloid PET and structural MRI data, and this index may potentially identify individuals with SCD with the AD-like pattern, as an appropriate risk population [183]. More comprehensively, Wirth et al. incorporated amyloid PET, FDG-PET and structural MRI data to determine the pathological pattern in the AD continuum. The results revealed three distinct imaging biomarker patterns, which were detected in individuals with different stages of AD [184].

Regarding the relationship between amyloid and functional alterations, several studies have presented diverse results. Chiesa et al. described an association between a greater amyloid load and reduced posterior basal forebrain resting-state functional connectivity (RSFC) in the hippocampus and thalamus [185]. Li et al. showed a positive association between a higher degree centrality [186] of the bilateral hippocampus and left fusiform gyrus with total tau and phosphorylated tau levels, rather than cerebral amyloid deposition [187]. Additional studies have reported significantly decreased WM connections and FC loss in individuals with SCD [113]. Thus, the relationship between AD pathology and brain function during the SCD stage lacks accurate evidence, which may be due to the different methodologies used to acquire parameters, preprocess data and quantify the results.

Multimodal studies involving EEG have indicated that the slowing property detected with EEG was related to white matter lesions (WMLs) and medial temporal atrophy (MTA), but not to the amyloid load [178, 188]. Gaubert et al. divided patients with SCD into four subgroups according to their amyloid status (based on ^18^F-florbetapir PET) and neurodegeneration status (based on FDG-PET). The results demonstrated that in neurodegeneration-positive subjects, amyloid burden was related to delta power following a U-shaped curve and related to other EEG metrics, such as gamma power, spectral entropy, and complexity, following an inverted U-shaped curve [189].

Tau protein deposition is regarded as another critical pathological biomarker of AD. However, the complicated relationships between amyloid, tau, neurodegenerations and cognitive decline are not clearly understood. Studies using amyloid PET and tau PET have coincidentally suggested that both tau protein and amyloid pathology contributed to the manifestation of SCD [190, 191]. Specifically, amyloid and tau pathologies may give rise to different subjective cognitive domains [191].

In longitudinal studies using FDG-PET and MRI modalities, the longitudinal reduction in cognitive performance was associated with brain hypometabolism in the precuneus at baseline, but not with gray matter atrophy [59]. Specifically, patients with SCD from the clinic displayed greater gray matter atrophy progression over time compared with patients with SCD from the community, indicating that clinical SCD may represent a greater risk of dementia due to AD [192]. Overall, the multimodal neuroimaging technique offers a great advantage in exploring the relationship between different AD biomarkers, and more multimodal neuroimaging studies of SCD are required (Table 5).

**Table 5:** Summary of multimodal studies

Authors	Definition of SCD	Modality	Design	Sample (mean age ± SD)	Main findings
Buckley et al. (2017) [190]	SCD composite questions	PiB-PET FTP-PET	Cross-sectional	All: *n* = 133 (75.9±7.0) Aß negative: *n* = 94 (74.9±7.2) Aß positive: *n* = 39 (78.4±5.7)	Greater SCD relate to increased entorhinal tau burden and Aß burden
Chetelat et al. (2010) [180, 181]	1 binary question	PiB-PET MRI	Cross-sectional	NC: *n* = 45 (74.9±7.1) SCI: *n* = 49 (73.9±7.2) MCI:*n* = 34 (75.4±7.2) AD: *n* = 35 (75.1±7.9)	Relation between global and regional atrophy and Aβ-amyloid load in SCI individuals but not in MCI or AD dementia
Che ´telat et al. (2010) [180, 181]	1 binary question	PiB-PET MRI	Cross-sectional	HC-: *n* = 32 (73.1±7.1) HC+: *n* = 13 (78.9±5.5) SCI-: *n* = 30 (72.1±7.1) SCI+: *n* = 19 (76.7±6.5) MCI+: *n* = 22 (75.8±7.1) AD+: *n* = 34 (75±7.9)	Larger temporal gray matter volume in HC with high amyloid load; gray matter atrophy in SCI with high amyloid load and MCI compared to HC
Chiesa et al. (2019) [140, 185]	2 binary questions	^18^F-florbetapir PET Rs-fMRI	Cross-sectional	Overall: *n* = 267 (75.8±3.5) *ApoE* ɛ4 noncarriers: *n* = 192 (75.7±3.6) *ApoE* ɛ4 carriers: *n* = 53 (76.1±3.6)	Higher SUVR values related to lower posterior basal forebrain RSFC in the hippocampus and the thalamus, impacted by sex and *ApoE* genotype
Chiesa et al. (2019) [140, 185]	2 binary questions	^18^F-florbetapir PET Rs-fMRI	Cross-sectional	All: *n* = 224 (75.5±3.4) *ApoE* ɛ4 noncarriers: *n* = 180 (75.5±3.4) *ApoE* ɛ4 carriers: *n* = 44 (75.6±3.5)	DMN changes in frontal and posterior areas and right hippocampus. No impact of brain amyloid load status on longitudinal RSFC.
Eliassen et al. (2017) [193]	Cognitive complaints	FDG-PET MRI	Cross-sectional	aMCI: *n* = 53(61.9±7.8) naMCI: *n* = 27(60.7±7.8) SCD: *n* = 38(59±8.3)	Lower cortical glucose metabolism in aMCI than SCD and controls. Thinner entorhinal cortex in SCD and aMCI
Ferreira et al. (2017) [183]	1 binary question	PiB-PET MRI	Cross-sectional	HC-like SMD: *n* = 75 (72.5±6.8) AD-like SMD: *n* = 11 (75.3±8.8)	The disease severity index identified eleven (13%) SCD with AD-like pattern of brain atrophy, who show lower cognitive performance, higher amyloid deposition, and worse clinical progression
Gaubert et al. (2019) [189]	Memory complaint	^18^F-florbetapir PET EEG	Cross-sectional	All: *n* = 314 (76.07±3.47) A-N-: *n* = 175 (75.62±3.39) A+N-: *n* = 63 (76.81±3.19) A+N+: *n* = 25 (76.88±4.01)	EEG metrics of fronto-central regions correlate with neurodegeneration. A U-shape or inverted U-shape relationships between amyloid burden and EEG metrics in neurodegeneration positive subjects
Kramberger et al. (2017) [188]	Memory clinic consultation	EEG MRI	Cross-sectional	SCI: *n* = 194 (57.7±7.5) MCI: *n* = 141 (61.7±8.3) AD: *n* = 58 (63.6±7.0)	WMLs and medial temporal atrophy relate to slower BA in all diagnoses
Kuhn et al. (2019) [192]	Composite of 10 questions CDS	^18^F-florbetapir PET FDG-PET MRI	Longitudinal (15-43 months)	HC: n=28 (72.25±6.33) SCD-community: *n* = 23 (71.70±6.60) SCD-clinic: *n* = 27 (68.30±7,99)	Higher self-reported SCD relate to lower gray matter volume and higher anxiety in SCD-community, to greater informant-reported SCD in SCD-clinic and to lower glucose metabolism in both SCD groups
Li et al. (2018) [187]	CCI	^18^F-florbetapir PET MRI	Cross-sectional	NC: *n* = 40 (75.10±5.39) SMC: *n* = 44 (73.78±5.81)	Higher DC in the bilateral hippocampus and left fusiform gyrus and lower DC in inferior parietal in SMC. DC in bilateral hippocampus and left fusiform relate to total tau and phosphorylated tau, but not to amyloid deposition
Scheef et al. (2012) [59]	Memory clinic consultation 2 binary questions	FDG-PET MRI	Cross-sectional	Controls: *n* = 56 (66.4±7.2) SMI: *n* = 31 (67.6±6.2)	Hypometabolism in right precuneus and hypermetabolism in right medial temporal and reduced gray matter volume in hippocampus in SMI group. Longitudinal memory decline relates to reduced glucose metabolism in right precuneus in SMI
Shokouhi et al. (2019) [191]	E-Cog	^18^F-flortaucipir PET ^18^F-florbetapir PET	Cross-sectional	All: *n* = 86 (78±8)	Tau pathology predict everyday planning in SCD, and amyloid pathology relate to everyday organization and memory in SCD
Teipel et al. (2018) [178]	2 binary questions	^18^F-florbetapir PET MRI EEG	Cross-sectional	Amyloid negative: *n* = 63 (75.9±3.5) Amyloid positive: *n* = 255 (76.7±3.5)	No significant relationship between amyloid load and phase-lag index in any frequency band
Teipel et al. (2017) [182]	2 binary questions	^18^F-florbetapir PET MRI	Cross-sectional	All: *n* = 318 (76.1±3.5)	Association between amyloid uptake and reduced gray matter structural integrity and poorer objective cognitive performance
Ten Kate et al. (2018) [101]	2 binary questions	^18^F-florbetapir PET MRI	Cross-sectional	All: n=318(76 74±78) Amyloid-: *n* = 230 (76 73±78) Amyloid+: *n* = 88 (77 75±79)	Association between higher global SUVR and lower clustering, and small world values in orbito-and dorsolateral frontal and parietooccipital regions.
Wirth et al. (2018) [184]	Memory clinic consultation	^18^F-florbetapir PET MRI FDG-PET	Cross-sectional	HC: n=41 (66.1 ±7.7) *ApoE* ɛ4+: *n* = 17 (63.9±8.6) SCD: n=16 (68.9±7.3) MCI: n=30 (73.4±7.2) AD: n=22 (68.7±9.4)	(1) in medial-temporal regions, local gray matter volume reduction exceeded hypometabolism, (2) in temporoparietal regions, hypometabolism predominated over gray matter volume reduction, and (3) in frontal regions, Aβ deposition exceeded gray matter volume reduction and hypometabolism. Three distinct biomarker patterns in MCI, only pattern 1 in SCD, only pattern 3 in *ApoE* ɛ4 carriers
Yasuno et al. (2015) [113]	EMC	PiB-PET MRI	Cross-sectional	nSCI: *n* = 30 (72.2±4.8) SCI: *n* = 23 (69.6±8.0)	Reduced FC in cortical midline structure in SCI. reduced WM connections relate to reduced FC. No amyloid deposition in SCI

*SCC* Subjective cognitive complaints, *ND* Neurodegeneration, *FDG* ^18^F-Fluorodeoxyglucose, *EEG* Electroencephalography, *WM* White matter, *SMD* Subjective memory decline, *FTP* Flortaucipir, *SCD* Subjective cognitive decline, *CDR* Clinical dementia rating, *E-Cog* Everyday Cognition Scale, *AD* Alzheimer’s disease, *SMI* Subjective memory impairment, *CMRglc* Cerebral metabolic rates for glucose, *ApoE Apolipoprotein E*, *FCSRT* Free and cued selective reminding test, *BNT* Boston naming test, *VOSP* Visual object and space perception battery, *ToL* Tower of London test, *IP* Isoprostane, *SUVR* Standardized uptake value ratio, *SCI* Subjective cognitive impairment, *MCI* Mild cognitive impairment, *aMCI* Amnestic MCI, *naMCI* Non-amnestic MCI, *NC* Normal control, *PET* Positron emission tomography, *PiB* Pittsburgh compound B. ADNI: Alzheimer’s Disease Neuroimaging Initiative, *MRI* Magnetic resonance imaging, *WMLs* White matter lesions, *rs-fMRI* Resting-state functional MRI, *DMN* Default mode network, *RSFC* Resting-state functional connectivity, *FC* Functional connectivity, *DC* Degree centrality

## Shortcomings and emerging trends

### Factors contributing to heterogeneous neuroimaging findings in SCD

The inconsistent and heterogeneous neuroimaging findings in SCD may result from several factors: (a) The use of different diagnostic criteria and assessment strategies for SCD may be a factor contributing to the heterogenous findings. Although unified research criteria for SCD have been proposed by SCD-I, it has not been universally used. The evaluation and classification measurements of SCD vary among investigations, including both qualitative methods (SCD/no SCD based on binary questions) and quantitative measures (e.g., E-Cog, MFQ, and Memory Assessment Clinics Questionnaire (MAC-Q)) (b) Variations in the demographics of the cohorts, both within and across studies, may be another influencing factor. Converging evidence has suggested that demographic characteristics such as age, sex, education level and the presence of the *ApoE* ɛ4 allele are important factors influencing cognition. However, the distributions of these demographics are highly variable. (c) Methodological differences in the acquisition of parameters and the quantification methods (e.g., voxel-based analysis, region-of-interest analysis, connectivity or connectome-based approaches) may also be factors producing some inconsistencies in the results, indicating that the interpretations and comparisons of these findings should be viewed with caution. However, despite the existence of these influencing factors, most studies included in the current review still described some common neuroimaging alterations in individuals with SCD.

### Longitudinal imaging studies

Longitudinal research in this field is still limited. Most of the studies investigating the neuroimaging changes in individuals with SCD often employ a cross-sectional design in which neuroimaging measures are compared between individuals with SCD and HCs. However, this commonly used design does not account for the differences in individual trajectories of brain changes. Longitudinal studies including follow-up scans enable the assessment of individual trajectories of brain changes and the identification of AD pathology in subjects with SCD. Additionally, longitudinal studies facilitate the investigation and validation of causality between pathological markers and emerging neurodegeneration and cognitive decline. Importantly, longitudinal designs allow researchers to explore biomarkers for the early prediction of disease conversion by investigating the subsets of patients with SCD who ultimately progress to dementia due to AD.

It is encouraging to learn that multiple international neuroimaging projects investigating dementia due to AD or preclinical AD are collecting data via longitudinal designs. Specifically, the ADNI database, an integral part of a multisite longitudinal study, is collecting multimodal imaging data (including MRI, DTI, fMRI and PET) and has started adding an SCD group from ADNI-2 [194]. MEMENTO is a clinic-based study that recruited 2323 patients with cognitive impairments and subjects with SCD at baseline who will be followed over a 5-year period [195]. In addition, the FACEHBI [196], INSIGHT-preAD [197], DELCODE [198] and SILCODE (trial registration: NCT03370744) [199] are ongoing longitudinal observational studies of individuals with SCD that will facilitate research exploring the developmental trajectory of different pathological biomarkers in AD. Importantly, opening and sharing these neuroimaging datasets of patients with SCD has been encouraged to accelerate the development of research in this field.

### Multimodal imaging studies

Different neuroimaging techniques have captured different aspects of the brain abnormalities involved in SCD to help reveal its multimodal signature. However, no single-modality imaging method is currently able to accurately characterize the pathological mechanisms underlying the full spectrum of SCD. Thus, the increasingly utility of multimodal neuroimaging technology provides an opportunity to determine the complicated relationships between amyloid, tau and downstream neurodegenerative pathologies occurring in the AD process. For SCD populations, several studies combining multimodal neuroimaging techniques such as PET and MRI have recently been conducted. However, the complicated relationships between distinct pathological biomarkers, such as amyloid, tau, and macroscale structural and functional brain alterations during the SCD stage, from local to connectivity level changes, still remain largely unexplored. More multimodal imaging studies are urgently needed to understand the interactions between different pathological changes in the early stage of AD.

Additionally, newly developed molecular tracers, imaging sequences and ultrahigh field MRI techniques, such as the use of 7-T scanners, will be helpful to detect more subtle alterations in the early stage of the disease and should be applied to further investigate SCD populations.

### Individual prediction with artificial intelligence

Artificial intelligence, such as machine learning and deep learning, offers a systematic approach to developing sophisticated, automatic, and objective classification frameworks for analyzing high-dimensional data. Additionally, artificial intelligence techniques are able to learn complex and subtle patterns of change across various imaging modalities [200]. Over the last decade, classification methods based on imaging have been increasingly integrated to identify the imaging signature of AD [201–203], offering promising tools for individualized diagnoses and prognostic predictions. However, until recently, neuroimaging-based studies for classifying SCD have been scarce [111, 126, 135, 204–206]. The early identification of SCD and the prediction of disease progression at the individual level is important for timely interventions. Furthermore, machine learning not only detects subtle and distributed changes but also enables the extraction of biomarkers from high-dimensional neuroimaging data. Recently, the neuroimaging-based “brain age” has been proposed as an important biomarker of an individual’s brain health [207]. Additionally, the SPARE-AD index was proposed based on a support vector machine (SVM) classifier between HCs and age-matched patients with dementia due to AD and was used to quantify the spatial pattern of abnormality [201]. Peter and colleagues used similar methods and showed that the extracted index was higher in individuals with SCD than in HCs [95]. The biomarker obtained based on machine learning might be more sensitive at detecting the early stage of AD because it captures a multivariable pattern. Overall, artificial intelligence combined with neuroimaging big data has the potential to enable individualized diagnoses of SCD due to AD and to extract sensitive imaging biomarkers from important features selected from high-dimensional neuroimaging data.

## Conclusions

In this review, we have provided a comprehensive summary of the molecular, structural and functional brain alterations of individuals with SCD related to AD investigated at different scales, ranging from regional to large-scale network-based imaging measures.

**Fig. 2:**
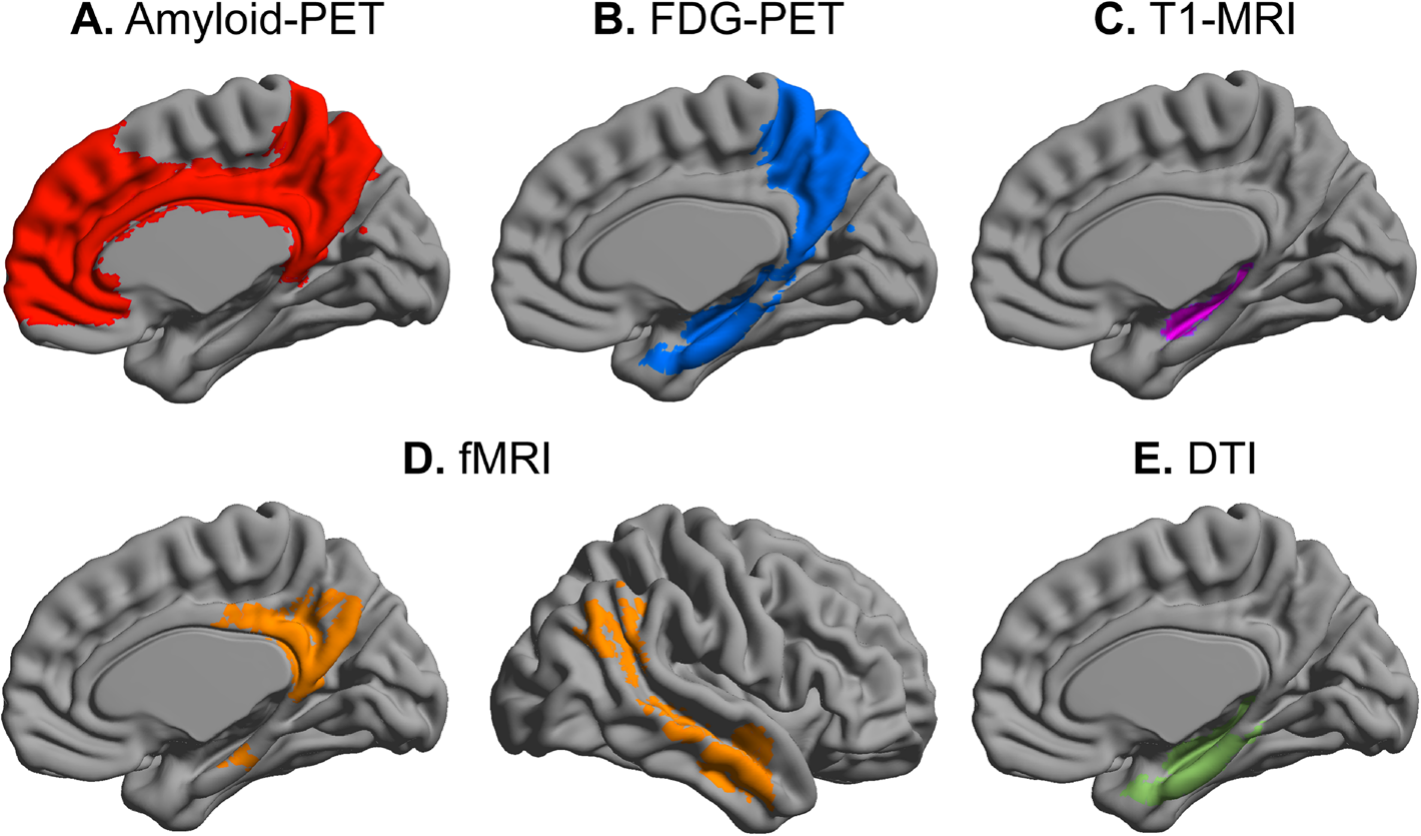
Multimodal imaging signature of SCD. The consistent results were collected from the articles included in this review to provide a comprehensive summary of common neuroimaging changes in SCD. **a** SCD individuals exhibit a pattern of amyloid accumulation within the medial prefrontal, cingulum and precuneus cortex, which are early sites of vulnerability in MCI and dementia due to AD. **b** The medial temporal lobe is frequently characterized by hypometabolism, whereas other studies have reported a strong association between SCD and hypometabolism within the precuneus. **c** Individuals with SCD showed decreased hippocampal volume and thinner entorhinal cortex than healthy controls. **d** The abnormal FC between the posterior DMN and other regions are frequently reported in resting-state fMRI studies. **e** DTI studies have reported decreased FA in hippocampal and parahippocampal white matter in SCD individuals. Abbreviations: SCD = Subjective cognitive decline; MCI = Mild cognitive impairment; AD = Alzheimer’s disease; DTI = Diffusion tensor imaging; DMN = Default mode network; fMRI = Functional MRI; FC=Functional connectivity

We collected consistent results from the articles included in this review and summarized the shared neuroimaging changes observed in individuals with SCD in the context of AD, as shown in Fig. 2. Regarding the pathological alterations at the molecular level, PET studies have observed early amyloid deposition, an increased tau burden and hypometabolism in individuals with SCD. MRI techniques enable assessments of alterations in macroscopic brain structures, such as decreased hippocampal volume and thinner entorhinal cortex; as well as microstructural deficits in WM tracts, such as decreased FA in the hippocampus and parahippocampal gyrus and abnormal functional activity. These assessments also illustrate the abnormal FC of the DMN and topological alterations in the whole-brain connectome. Based on these findings, we identify a preferential vulnerability of highly selected brain regions that are mainly affected in individuals with MCI or dementia due to AD, including the hippocampus, medial temporal lobe, precuneus and temporoparietal regions, indicating that individuals with SCD share a similar pattern of pathological alterations with individuals with MCI and dementia due to AD. As different neuroimaging techniques can reflect different aspects of brain abnormalities, we also suggest that the combination of multiple imaging modalities may provide a more comprehensive understanding of the pathological process than a single modality. However, a small number of conflicting findings of neuroimaging changes in individuals with SCD due to AD exist, including reports of no relationship between SCD and amyloid pathology, the preservation of gray matter structure (e.g., the hippocampal volume) and WM integrity, and even hypermetabolism of cerebral glucose in SCD subjects. In particular for studies of brain function, although most studies have reported abnormal FC of the DMN in individuals with SCD, the directions of these results (i.e., increased FC or reduced FC) are still relatively inconsistent. These inconsistent results may be due to the differences in the methods used to classify and assess SCD, the demographics of the cohorts, and the acquisition of parameters and quantification methods.

In conclusion, the pathological alterations underlying the manifestation of SCD are parallel to those underlying MCI and dementia due to AD based on the results obtained using neuroimaging techniques, supporting the notion that SCD represents an early precursor of dementia due to AD. While cognitive function is preserved, early detection of SCD is imperative to ensure that patients will benefit from early intervention and obtain the appropriate treatment in a timely manner. In the future, with additional validation studies of larger samples and longitudinal studies, the combination of multimodal neuroimaging techniques may help identify SCD individuals presenting with early AD pathologies who may be eligible for clinical trials for the early detection and secondary intervention of AD.

## Data Availability

Not applicable.

## Abbreviations

SCD: Subjective cognitive decline
AD: Alzheimer’s disease
PET: Positron emission tomography
MRI: Magnetic resonance imaging
*ApoE*: *Apolipoprotein* E
MFQ: Mood and Feelings Questionnaire
E-Cog: Everyday Cognition Scale
SCD-I: Subjective Cognitive Decline Initiative
PiB: Pittsburgh Compound B
HCs: Healthy controls
FDG: ^18^F-Fluorodeoxyglucose
MVPA: Multivariate pattern analysis
DTI: Diffusion tensor imaging
WM: White matter
FA: Fractional anisotropy
MD: Mean diffusivity
RD: Radial diffusivity
fMRI: Functional MRI
BOLD: Blood oxygen level-dependent
rs-fMRI: Resting-state fMRI
DMN: Default mode network
ALFF: Amplitude of low-frequency fluctuations
FC: Functional connectivity
ASL: Arterial spin labeling
CBF: Cerebral blood flow
EEG: Electroencephalography
MEG: Magnetoencephalography
RSFC: Resting-state functional connectivity
WMLs: White matter lesions
MTA: Medial temporal atrophy
SVM: Support vector machine
MAC-Q: Memory assessment clinics questionnaire
CSF: Cerebrospinal fluid
MCI: Mild cognitive impairment
ADNI: Alzheimer’s Disease Neuroimaging Initiative
